# Emergency vaccination alleviates highly pathogenic porcine reproductive and respiratory syndrome virus infection after contact exposure

**DOI:** 10.1186/1746-6148-9-26

**Published:** 2013-02-09

**Authors:** Xiao Li, Li Qiu, Zengqi Yang, Ruiyi Dang, Xinglong Wang

**Affiliations:** 1 College of Animal Science and Technology, Northwest A&F University, Yangling, 712100, China; 2 College of Veterinary Medicine, Northwest A&F University, Yangling, 712100, China

## Abstract

**Background:**

To assess the effectiveness of emergency vaccination for reducing the contact-induced infection and pathological damage caused by the highly pathogenic porcine reproductive and respiratory syndrome virus (HPPRRSV), Twenty pigs were equally divided into four groups. Groups 1, 2 and 3 were housed in one unit, whereas Group 4 was separately housed. Group 1 was challenged with HPPRRSV on day 0. Group 2 and 4 did not receive treatment and were used as the contact-infected and uninfected controls, respectively. Group 3 was treated with the attenuated vaccine at 0 days post-inoculation. The rectal temperatures, clinical signs, pathologic lesions and viraemia of the piglets were detected and evaluated.

**Results:**

The vaccinated pigs in Group 3 showed less clinical morbidity, viraemia, temperature fluctuations and lung lesions at 14 days post-inoculation, as compared with the contact-infected (Group 2) and experimentally infected (Group 1) pigs. Higher serum IFN-γ levels were detected among the pigs that received emergency immunisation. Thus, IFN-γ may be involved in immunity against HPPRRSV infection.

**Conclusions:**

These results indicated that emergency vaccination could effectively alleviate HPPRRSV infection during experimental contact exposure. Our findings provide a novel and useful strategy for controlling clinical HPPRRSV.

## Background

The highly pathogenic porcine reproductive and respiratory syndrome virus (HPPRRSV) in China was first reported in 2006; the outbreak overwhelmed ten provinces (including autonomous cities or regions) with more than 2,000,000 infected pig within the first four months [1]. HPPRRSV was likewise reported in Vietnam, where it caused much economic loss to local farms [2]. Thus, HPPRRSV has emerged as one of the most important pathogens that threaten pig farms.

An HPPRRSV-derived attenuated vaccine was developed to control the disease [3]. The attenuated vaccine of a modified-live virus (MLV) derived from the American PRRSV VR-2332 has been widely used in PRRSV-prevalent countries, with its safety and effectiveness proven by previous studies [4,5]. However, clinical observations showed that several MLV-vaccinated farms in China suffered from heavy economic losses caused by HPPRRSV in 2006 to 2010 [6]. This inconsistency revealed that the MLV vaccine provides limited protection from HPPRRSV under normal immunisation procedures.

Several farms in Jiangsu, China successfully reduced HPPRRSV damage using a promising emergency immunisation strategy with the MLV strain. An excess dose of the MLV vaccine (4 to 6 doses) was administered upon confirmation of HPPRRSV infection. Losses were reduced by 30% to 70%, as compared with the untreated herds (unpublished data). Vaccine intervention against typical PRRSV has been previously studied. Although not as effective as a cure, vaccine intervention could reduce the persistence and transmission of PRRSV in a pig population infected with the heterogonous isolates [5,7].

This study aimed to replicate clinical cases under experimental conditions to confirm the effects of emergency immunisation, which may be widely used for emergency cases of HPPRRSV infection.

## Methods

### Virus

The Northern American PRRSV isolate BB0907 was obtained from infected pigs in 2009, purified, and passaged using MARC-145 cells. The BB0907 isolate (9th passage on MARC-145) is highly virulent and caused high mortality in piglets in previous experimental infection experiments [8]. The widely used vaccine Ingelvac&reg PRRS MLV was purchased from Ingelvac.

### Animals

A total of 20 PRRSV-free crossbred (Landrace × local stock) pigs, approximately 28-days-old, were randomly distributed into four groups. Groups 1, 2 and 3 were housed in one unit, whereas Group 4 was housed in another.

All experimental procedures were approved by an independent animal care and use committee. The guidelines of the National Veterinary Research and Quarantine Service for the reproduction of pathogenesis in pigs were respected.

### Infection and immunisation

The Group 1 pigs were intramuscularly injected with 2×10^4^ TCID_50_/ml BB0907 in 2 ml Dulbecco’s modified Eagle’s medium at 0 day post-inoculation (DPI). The Group 3 pigs were intramuscularly vaccinated with three doses of Ingelvac® PRRS MLV (10^5^ TCID_50_/ml) at 0 DPI. The pigs in the Groups 2 and 4 did not receive any treatment.

### Clinical and pathologic examination

Rectal temperatures, clinical signs, pathologic lesions and viraemia were detected and evaluated following the procedures of our previous study [9]. Sera were collected at 0, 3, 5, 7, 10, 14 and 21 DPI to detect the virus load, serum IFN-γ concentration and PRRSV-specific antibody. At 3, 5, 7, 14 and 21 DPI, the following clinical signs were graded using a scale from 0 to 1: anorexia, lethargy, rough hair, dyspnoea and cough. Gross lung lesions (0 to 2 points) were evaluated based on gray mottling, oedema and consolidation. The severity of haemorrhage and the enlargement of lymph nodes were scored using three grades (0 to 2 points). All pigs were euthanised and necropsied on 21 DPI. Lung sections for histopathologic examination were collected and prepared, as previously described [10]. Lung histopathology was determined in terms of the degree of haemorrhage (0 to 1 point) and interstitial pneumonitis (0 to 1 point). The highest pathologic lesion score for the morbid pigs was 12 (total score of all the pathologic lesions).

### Antibody measurement

Enzyme-linked immunosorbent assay (ELISA) S/P (Sample/Positive) ratios were determined using the HerdCheck® PRRS ELISA 2×R (IDEXX Laboratories, Inc., Westbrook, ME, USA) according to the manufacturer’s instructions. The sera were collected on 0, 3, 5, 7, 10, 14 and 21 DPI.

### Serum IFN-γ concentration

To detect IFN-γ production in pigs, serum was collected at 0, 3, 5, 7, 14 and 21 DPI for quantitative measurement of IFN-γ using commercial ELISA kits (R&D Systems, Minneapolis, MN, USA), according to the recommended protocol. The test had three replicates for each sample, and the data were presented as the mean ± S.E.

### Viraemia detection

Viraemia was determined at 0, 3, 5, 7, 14 and 21 DPI by real-time polymerase chain reaction amplification using HPPRRSV-specific primers, as previously described [11]. To quantify the serum virus load, cDNA from cultured PRRSV (with a known TCID_50_) was serially diluted by tenfold to generate a standard curve. The amount of virus in the samples was determined by linear extrapolation of the *Ct* value plotted against the standard curve.

### Statistical analysis

All data are presented as mean ± S.D. *χ*^2^-test was used to analyze the clinical signs and gross lesions of the animals after challenge. The cytokines and viremia data were evaluated using one-way repeated measurements ANOVA and least significance difference. Differences with *p < 0.05* were considered statistically significant.

## Results

### Clinical signs and pathologic examination

**Figure 1:**
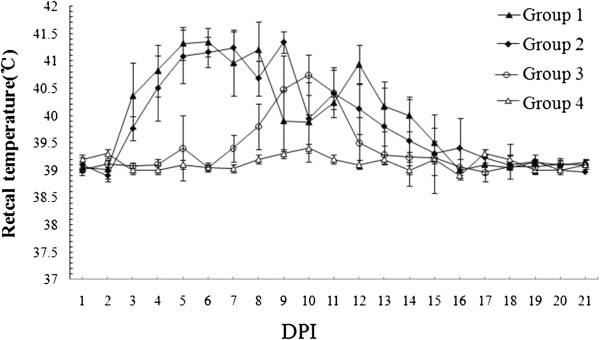
**Rectal temperature of the pigs with different treatment.** The data was presented as the mean ± S.D.

All pigs exposed to BB0907, except those in Group 3, exhibited high fever (≥ 41°C) for more than five days (Figure 1). The pigs in Group 3 had slight temperature variations, with 2 pigs from this group experiencing a three-day fever of approximately 40.5°C. Aside from the high fever, the appearance of typical HPPRRSV-induced characteristics was delayed for almost eight days in Group 3. As expected, the pigs in Group 4 had no relevant temperature changes throughout the duration of the experiment.

The clinical signs of HPPRRSV infection were observed at 3, 5, 7, 10, 14 and 21 DPI; these included anorexia, lethargy, reddened skin, dyspnoea and cough. The pigs in Groups 1 and 2 exhibited anorexia, lethargy and reddened skin from 5 DPI to 7 DPI. Among the pigs in these groups, three had dyspnoea and cough at 7 DPI. At 10 DPI, 3 pigs in Group 1 and 2 pigs in Group 2 had died. By contrast, the pigs in Group 3 only demonstrated slight lethargy, and none of the individuals died during the experiment. The pigs in Group 4 likewise exhibited no clinical signs of infection. Significantly serious tissue lesions were observed in Groups 1 and 2, as compared with those in Groups 3 and 4. These results are summarised in Table 1.

**Table 1:** Summary of infection and treatment results from piglets

			**Scores of clinical signs of each pig**					Scores of pathological lesions of each pig***					
**Groups**	Morbidity*	Mortality**	**3dpi**	**5dpi**	**7dpi**	**14dpi**	**21dpi**	**1#**	**2#**	**3#**	**4#**	**5#**	**Mean ± SD**
Group1	5/5	3/5	1.6	2.2	3.2	4.5	3.2	8	12	9	12	12	10.6±1.83^a^
Group2	5/5	2/5	1	2	2.4	3	2.25	7	8	12	12	7	9.2±2..4^a^
Group3	3/5	0/5	0	1.4	0.4	3.2	1.6	5	7	4	9	7	6.40±1.58^b^
Group4	0/5	0/5	0	0	0	0	0	1	2	0	0	0	1.00±0.89^c^

* indicates numbers of ill pigs/total numbers of pigs within a group;

** indicates numbers of died pigs/total numbers of pigs within a group;

*** indicates mean scores of the clinical signs of living pigs;

# labels the ear number of pigs within one group;

The different labeled letter “a,b,c” in the last line indicates significant difference between different groups (*P* < 0.05).

**Figure 2:**
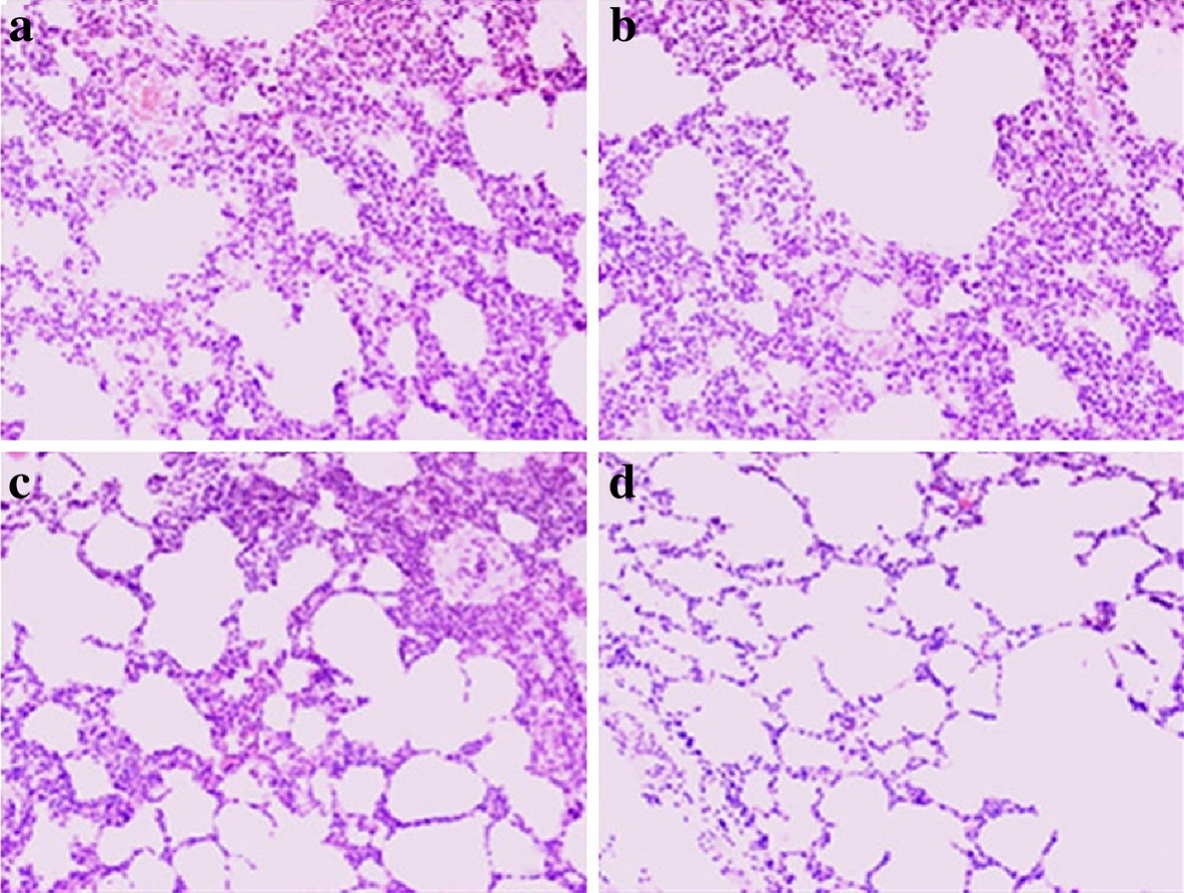
**Microscopic examination of infected lungs compared with the healthy ones on 21 DPI.** Hematoxylin- and eosin- stained sections of lungs in pigs from Group1 (**a**), Group 2 (**b**), Group 3 (**d**) and Group 4 (**c**) on 21 DPI. Images were obtained on an Olympus BX-50 light microscope at 200-fold original magnifications.

The euthanised pigs were necropsied, during which their lungs and lymph nodes were examined. The results showed that the most serious gross lesions were observed in Groups 1 and 2, including gray mottling, oedema, lung consolidation, haemorrhage and lymph node enlargement. Only a few cases of minor gray mottling were observed in Group 3, with very slight signs of oedema and lung consolidation. The lungs of pigs in Group 4 were relatively clean, with one pig having a slight congestion. Microscopic lesions in the lungs were evaluated in terms of their septal thickening and haemorrhage. Lungs of the virus-inoculated pigs showed microscopic lesions characterised by some septal thickening and haemorrhage (Figure 2). Lungs collected from Groups 1 and 2 showed severe haemorrhage and septal thickening, whereas lungs from Group 3 only exhibited slight lesion. The lungs obtained from Group 4 were relatively healthy (Table 1).

### Antibody measurement

**Figure 3:**
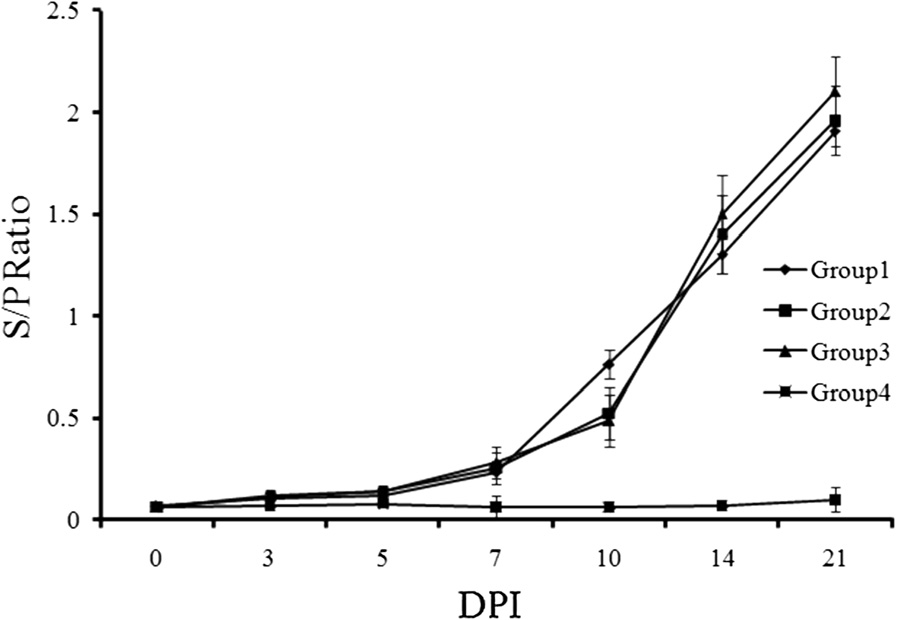
**Kinetics of antibody response to PRRSV detected by commercial available ELISA kit.** The serum samples (n=5) were collected from 0 to 21 DPI as indicated. Data were presented as the mean value of triplicate samples ± S.D.

The humoral immune response to PRRSV measured by the ELISA S/P ratios at 10 DPI showed that the average antibody titres of the virus-exposed groups exceeded the 0.4 cut-off for a positive result. By contrast, the control group averages were negative and remained below 0.4 until 21 DPI. No significant differences were found among the first three groups (Figure 3).

### Viraemia

**Figure 4:**
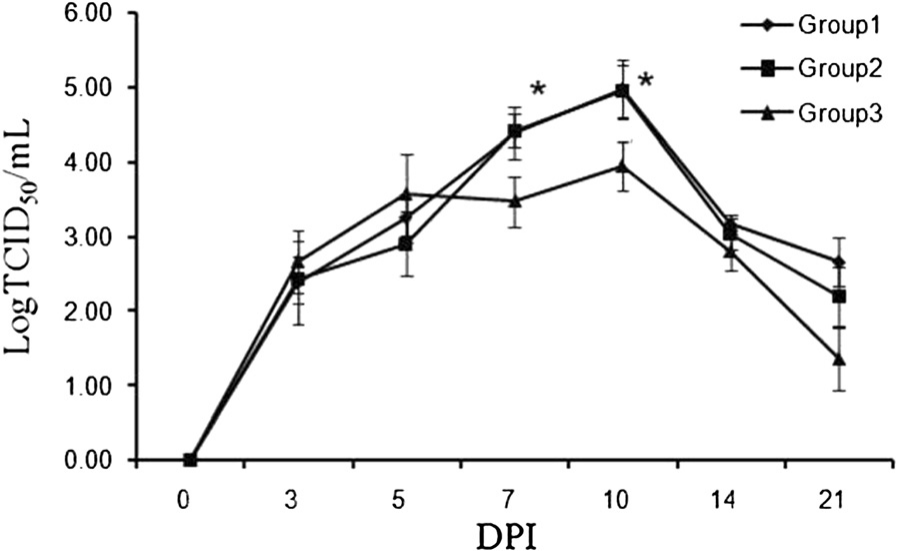
**Detection of the virus load in sera of the pigs by SYBR Green real-time PCR after challenge.** Serum samples were collected from living pigs in each group at different days post infection. Data were presented as the mean value of triplicate samples ± S.D. * means significant difference between Group 2 and 3*(P<0.05).*

The viraemia test demonstrated that the level of PRRSV transcripts in Groups 1 and 2 were significantly higher than that in Group 3 at both 7 and 10 DPI (*p < 0.05*) (Figure 4). A study to detect persisted infection in emergency immunised pigs should be conducted in future. PRRSV transcripts were not detected in Group 4 (*Ct* ≥ 40, data not shown).

### IFN-γ secretion

**Figure 5:**
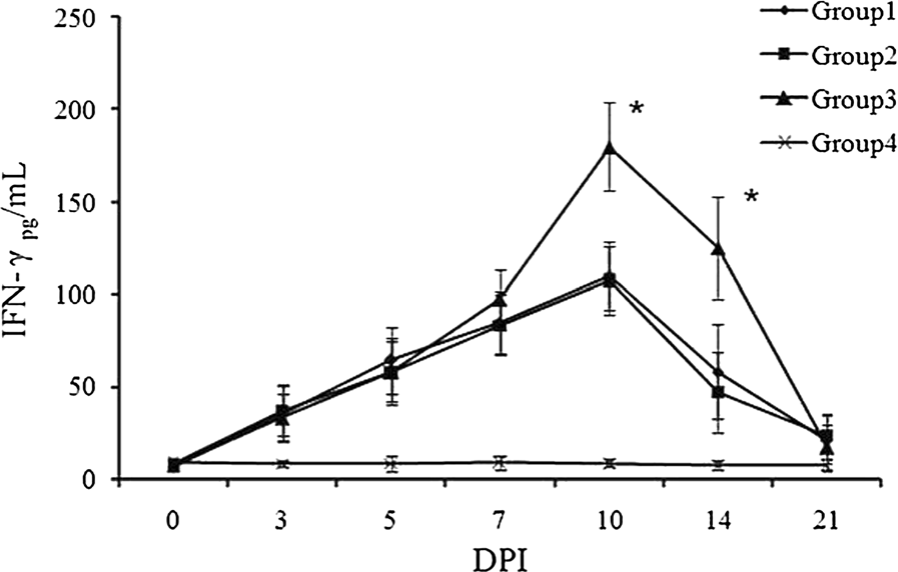
**Sera IFN-γ levels detected by commercial ELISIA kit.** The sera were collected from the pigs (n=5) at 0, 3, 5,7, 10, 14 and 21 DPI. Data were presented as the mean value of triplicate samples ± S.D. * means significant difference between Group 2 and 3 *(P<0.05).*

High IFN-γ serum levels were observed in the pigs exposed to the virus. The highest serum IFN-γ concentration was observed in Group 3, which was administered the emergency vaccination. The higher IFN-γ levels lasted for approximately 14 DPI, and the peak was observed at 10 DPI (Figure 5).

## Discussion

Emergency vaccination is a useful tool for controlling animal and human infectious diseases after exposure to pathogens, such as the foot-and-mouth disease virus (FMDV) [12], the classical swine fever virus (CSFV) [13], and rabies virus [14]. Emergency vaccination diminishes economic losses by reducing morbidity and mortality, as well as virus transmission. Post-exposure vaccination for typical PRRS has significantly reduced the number of persistently infected pigs at 127 DPI and reduced viral shedding to within 97 DPI [5]. In the present study, emergency vaccination successfully alleviated the clinical signs of HPPRRSV infection and reduced the mortality rate. Emergency vaccination was more efficient in controlling HPPRRSV, an acute form of the disease with epidemiologic characteristics that differed from typical PRRS.

The mechanism for emergency vaccination may be related to a quick adaptive immune response to restrict viral replication and proliferation, which could explain the immune protection conferred by the C strain of CSFV [13] and FMDV emergency vaccine [12]. Emergency vaccination might induce innate immunity. During rabies vaccination, the attenuated rabies virus spreads from the peripheral sites of inoculation to the CNS tissues, and triggers the substantial immune cell infiltration into the CNS. These cells had a major function in the early containment of rabies viral infections (i.e., cleaning rabies or preventing them from entering the CNS), particularly through the production of type I interferon [14]. If the attenuated rabies vaccines entered the CNS after the wild-type rabies virus, the vaccination would be ineffective [14,15]. Previous studies have provided very little explanation on the mechanism of PRRSV emergency vaccination. PRRSV variants possess different capacities for inducing or controlling innate immunity, which appears similar with rabies vaccination. Thus, we speculated that the attenuated PRRSV might trigger an innate immune response that subsequently controls HPPRRSV infection.

The significantly higher level of serum IFN-γ in the vaccine-treated group at 10 DPI lasted until the end of the experiment (21 DPI). This process probably influenced the protection obtained by piglets from the vaccinated group. In a previous report [16], a swine serum IFN-γ response was detected immediately after PRRSV infection and lasted for approximately 3 weeks. IFN-γ is important for controlling PRRSV infection [17]. IFN-γ could inhibit PRRSV replication more effectively than the type I interferon *in vitro*[18,19]. Furthermore, no neutralizing activities were detected in all of the serum samples (data not shown). This observation underlines the protective function of IFN-γ during the early stages of PRRSV infection.

The viral load in tissues of infected pigs under the acute infection phase was one of indexes used to indicate PRRSV pathogenicity. The more virulent the strain is, the higher is the viral load in pigs [17]. In our study, the serum viral RNA load was significantly lower in the vaccinated group (*p < 0.05*) at 7 and 10 DPI. The severity of a clinical disease is highly associated with the viral load [20]. Thus, the lower serum viral RNA load might account for the minimal clinical signs and tissue lesions observed in the vaccinated group.

In this study, we developed an HPPRRSV contact-infection model by intramuscular infection. The infected pigs exhibited higher levels of viraemia at 3 DPI, thereby suggesting that the pigs transmitted the virus within 2 DPI, which may account for the rapid spread of the virus in herds [21]. PRRSV transmission is primarily via the respiratory route [22]. Our data indicated that the virus from the inoculation sites rapidly reached the lungs.

## Conclusions

To the best of our knowledge, this is the first report that uses emergency vaccination to control HPPRRSV infection. The experiment demonstrated a reduction in viraemia by approximately 90% at 7 DPI to 10 DPI. The tissue lesions scores ranged from 10.6 to 6.4 and the mortality from 3/5 to 0/5 during the HPPRRSV contact-infection. These data may provide a useful reference for future methods of clinical HPPRRSV control.

## Competing interest

The authors declared that they have no competing interests.

## Authors’ contributions

XL and LQ carried out the animal assays, the immunoassays and drafted the manuscript. ZY and RD conceived of the study, and participated in its design and coordination and helped to draft the manuscript. XW participated in the design of the study and performed the statistical analysis. All authors read and approved the final manuscript.
